# Potentiometric Aptasensing of *Vibrio alginolyticus* Based on DNA Nanostructure-Modified Magnetic Beads

**DOI:** 10.3390/s16122052

**Published:** 2016-12-02

**Authors:** Guangtao Zhao, Jiawang Ding, Han Yu, Tanji Yin, Wei Qin

**Affiliations:** 1 Key Laboratory of Coastal Environmental Processes and Ecological Remediation, Yantai Institute of Coastal Zone Research (YIC), Chinese Academy of Sciences (CAS); Shandong Provincial Key Laboratory of Coastal Environmental Processes, YICCAS, Yantai 264003, Shandong, China; gtzhao@yic.ac.cn (G.Z.); jwding@yic.ac.cn (J.D.); hyu@yic.ac.cn (H.Y.); 2 University of Chinese Academy of Sciences, Beijing 100049, China

**Keywords:** potentiometric aptasensing, *Vibrio alginolyticus*, DNA nanostructures, magnetic beads, protamine

## Abstract

A potentiometric aptasensing assay that couples the DNA nanostructure-modified magnetic beads with a solid-contact polycation-sensitive membrane electrode for the detection of *Vibrio alginolyticus* is herein described. The DNA nanostructure-modified magnetic beads are used for amplification of the potential response and elimination of the interfering effect from a complex sample matrix. The solid-contact polycation-sensitive membrane electrode using protamine as an indicator is employed to chronopotentiometrically detect the change in the charge or DNA concentration on the magnetic beads, which is induced by the interaction between *Vibrio alginolyticus* and the aptamer on the DNA nanostructures. The present potentiometric aptasensing method shows a linear range of 10–100 CFU mL^−1^ with a detection limit of 10 CFU mL^−1^, and a good specificity for the detection of *Vibrio alginolyticus*. This proposed strategy can be used for the detection of other microorganisms by changing the aptamers in the DNA nanostructures.

## 1. Introduction

As an opportunistic marine pathogen, *Vibrio alginolyticus* (*V. alginolyticus*) can not only lead to septicemias and corneal opaqueness in fish and shell diseases and white spots in shrimps [1,2,3], but also infects mammalian cells causing otitis, wound infection, and chronic diarrhea [4,5]. The rapid and sensitive detection of *V. alginolyticus* is of great importance for aquaculture, food industry, and clinical diagnosis.

The agar plate culture method and enzyme-linked immunosorbent assays are commonly used for the detection of bacteria. However, they suffer from such problems as time-consuming procedures, false-positive signals, low sensitivity, and poor specificity [6]. The polymerase chain reaction (PCR) technique, especially for the multiplex PCR, has been developed for the detection of bacteria with good specificity and high sensitivity [7,8,9,10]. However, this technique needs complex pretreatment procedures and expensive instruments.

Aptamers are single-stranded DNA or RNA oligonucleotides designed through the systematic evolution of ligands by exponential enrichment. Since aptamers have high specificities and binding affinities to their targets, they have been widely used to develop a variety of electrochemical biosensors for the detection of small molecules, metal ions, and proteins [11,12,13,14,15]. Nowadays, aptamers have also been used for the detection of bacteria based on their high specificity bindings to proteins either on the cell surfaces or inside the cells [16,17,18,19]. Various electrochemical aptasensors using different transduction modes such as amperometry, impedimetry, and potentiometry have been developed [20,21,22]. Recently, our group developed a label-free potentiometric aptasensing method for the detection of *Listeria monocytogenes* in a homogeneous solution [23], based on the polycation-sensitive membrane electrode using protamine as an indicator. However, for that method, other bacteria with negative charges may interfere with the potential response of protamine. Additionally, the polycation-sensitive membrane electrode may suffer from problems of strong potential drifts, since the extraction of polycation into the membrane is an irreversible process. The purpose of this work was to develop a new method for the potentiometric detection of bacteria, which could not only eliminate sample interferences but also show stable and reversible potential responses.

Herein, we present a novel potentiometric aptasensing strategy for the detection of *V. alginolyticus* based on DNA nanostructure-modified magnetic beads for signal amplification and magnetic separation, and on a solid-contact polycation-sensitive membrane electrode for reversible potential detection via chronopotentiometry. In the presence of *V. alginolyticus*, the DNA nanostructure-modified magnetic beads can be disassembled, which is induced by the specific binding interactions between the target bacterial cells and the aptamer molecules in the DNA nanostrutures. After magnetic separation, the change in the charge or DNA concentration on the magnetic beads can be chronopotentiometrially detected by the solid-contact polycation-sensitive electrode using protamine as an indicator based on the electrostatic interaction between DNA and protamine.

## 2. Materials and Methods

### 2.1. Materials 

High-molecular-weight poly(vinyl chloride) (PVC), 2-nitrophenyl octyl ether (*o*-NPOE), tetradodecylammonium chloride (TDDACl), dinonylnaphthalene sulfonic acid (DNNS, 50 wt % solution in heptanes), tris (hydroxymethyl)-aminomethane (Tris), protamine sulfate salt, and ionic liquid (IL, 1-butyl-3-methyl-imidazolium tetrafluoroborate) were purchased from Sigma-Aldrich. Carboxylic-multiwall carbon nanotubes (MWCNTs) were obtained from Nanjing XFNANO Materials Technology Co. Ltd. (Nanjing, China). The lipophilic salt DNNS-TDDA was synthesized as described before [24]. The magnetic beads (1 μm in diameter) were purchased from Bio Canal Co. Ltd. (Wuxi, China). Bacterial strains for *V. alginolyticus* 1.1587 and *Aeromonas hydrophila* 1.172 were purchased from Yudingxinjie Technology Co. Ltd. (Beijing, China). Bacterial strains for *Escherichia coli* (*E. coli*) ATCC 27853 and *Staphylococcus aureus* 08032813 were kindly provided from the Biotechnology Lab of Binzhou Medical University. The number of colony-forming units per mL (CFU mL^−1^) for each bacteria culture was determined by the surface plate counting method. The aptamer used in this work for the detection of *V. alginolyticus* was selected by Zheng et al. [3]. The sequences of the aptamer DNA and other DNA are shown in Table 1. All the sequences were synthesized by Shanghai Sangon Biotechnology Co. Ltd. (Shanghai, China). The buffer for DNA hybridization contained 100 mM NaCl, 5 mM KCl, 50 mM Tris-HCl, and 1 mM MgCl_2_ (pH = 7.4).

### 2.2. Synthesis of the DNA Nanostructure-Modified Magnetic Beads

All the oligonucleotides were kept at 95 °C for 2 min and then cooled at room temperature for 1 h before use [25,26,27]. The DNA nanostructure-modified magnetic beads were prepared as described before [28]. The capture DNA modified magnetic beads were obtained by incubating 150 μL of 5’-biotin-modified capture DNA fragments (3 μM) with 150 μL of streptavidin modified magnetic beads (10 mg·mL^−1^) via shaking for 0.5 h. After magnetic separation and washing with buffer, the capture DNA modified magnetic beads were incubated with 150 μL of aptamer DNA fragments (3 μM) via shaking for 1 h, and the capture/aptamer DNA modified magnetic beads were thus obtained. The H1/H2 DNA complexes were prepared by incubating 150 μL of H1 DNA fragments (3 μM) with 150 μL of H2 DNA fragments (3 μM) via shaking for 1 h. The H1/H2 DNA complexes (150 μL) were then incubated with 150 μL of capture/aptamer DNA modified magnetic beads with 1 h shaking. After magnetic separation and washing with buffer, the DNA nanostructure-modified magnetic beads were obtained and re-dispersed in 150 μL of buffer solution. For the detection of bacteria, 40 μL of the prepared DNA nanostructure-modified magnetic bead solution (10 mg·mL^−^^1^) were used.

### 2.3. Preparation of the Solid-Contact Polycation-Sensitive Electrode

Prior to modification, the glassy carbon electrode (GCE, 3 mm in diameter) was polished with emery paper and alumina slurries followed by rinsing thoroughly with ultrapure water. The electrode was successively ultrasonicated in water and ethanol, and then allowed to dry at room temperature.

A mixture of 5 mg of MWCNTs and 1 μL of IL was ground in an agate mortar for 10 min [29], and then dispersed in 1 mL of ultrapure water with ultra-sonication to obtain a 5 mg·mL^−1^ suspension solution. Twenty microliters of the MWCNT-IL suspension solution was coated onto the surface of the GCE and allowed to dry under an infrared lamp.

The polycation-sensitive membrane contained 10.0 wt % DNNS-TDDA, 60.0 wt % *o*-NPOE, and 30.0 wt % PVC. One hundred milligrams of the membrane components were dissolved in 800 μL of tetrahydrofuran (THF). Eighty microliters of the membrane solution were coated onto the MWCNT-IL modified GCE. After the evaporation of THF, the prepared solid-contact polycation-sensitive electrodes were conditioned in 10 mM NaCl for at least 12 h before use, and kept in the conditioning solution when not in use.

### 2.4. Determination of V. alginolyticus via Chronopotentiometry

Forty microliters of DNA nanostructure-modified magnetic beads were mixed with 50 μL of *V. alginolyticus* at different concentrations at room temperature for 30 min. After magnetic separation, the resulting magnetic beads were then incubated with 7.5 μL of 1 mg·mL^−1^ protamine. The unreacted protamine after magnetic separation was added into 1.5 mL of 0.01 M NaCl and detected by the solid-contact polycation-sensitive electrode via chronopotentiometry.

Chronopotentiometry was performed by using a CHI-760C electrochemical workstation (Chenhua Corp., Shanghai, China) with a conventional three-electrode system comprising the proposed solid-contact polycation-sensitive membrane electrode as a working electrode, a platinum wire as counter electrode, and an Ag/AgCl electrode (saturated KCl) as a reference electrode. During the chronopotentiometric experiments, an external cathodic current of 5 μA with a duration of 1 s was used to extract protamine into the polycation-sensitive membrane. The solid-contact polycation-sensitive membrane electrode was refreshed under a controlled voltage at the open-circuit potential in the absence of protamine with a recovery time of 180 s for multiple consecutive measurements. The difference between the potentials measured at 0.5 s after applying the current in the absence and presence of *V. alginolyticus* was used for quantification.

## 3. Results and Discussion

### 3.1. Sensing Principle

**Figure 1:**
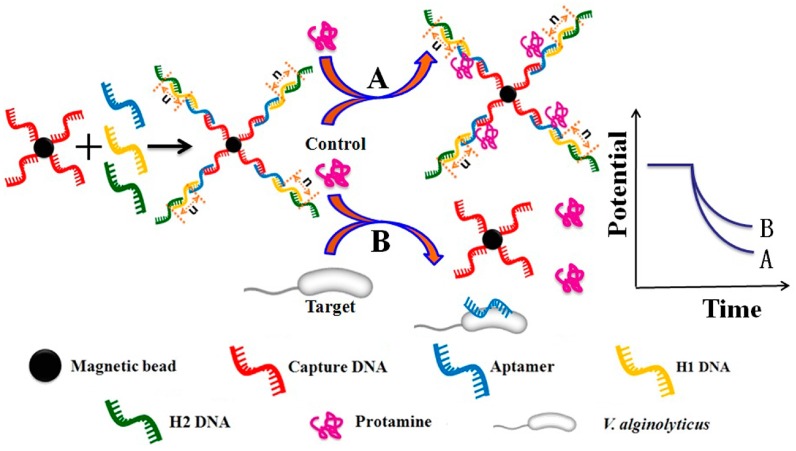
Schematic illustration of potentiometric aptasensing of *V. alginolyticus* based on DNA nanostructure-modified magnetic beads for (**A**) a control and (**B**) a given target assay.

Figure 1 shows the principle for the potentiometric aptasensing of *V. alginolyticus* based on DNA nanostructure-modified magnetic beads. The capture DNA was immobilized on the surface of the magnetic beads via the strong streptavidin-biotin interaction. The aptamer and H1/H2 DNA hybridize successively with the capture DNA through the complementary base-pairing reactions to form the DNA nanostructure-modified magnetic beads. In the presence of the target, the aptamer on the DNA nanostructure-modified magnetic beads specifically binds to the target, which leads to the disassembly of the DNA nanostructures and subsequently influences the charge or DNA concentration on the magnetic beads [28]. This aptamer/target binding event can be detected by the solid-contact polycation-sensitive electrode using protamine as an indicator, based on the electrostatic interaction between the DNA nanostructures and protamine.

**Figure 2:**
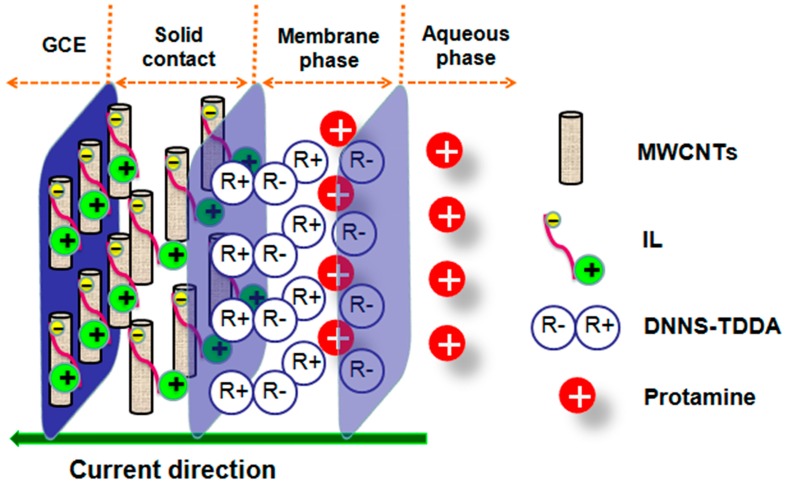
Schematic diagram of the polycation-sensitive electrode based on a MWCNT-IL composite as a solid contact for the chronopotentiometric detection of protamine.

Figure 2 shows the principle for the detection of protamine with the polycation-sensitive electrode based on the MWCNT-IL composite as solid contact via chronopotentiometry. The application of the imidazolium-based IL is to prevent the formation of agglomerates of MWCNTs through cation-π interactions between the imidazolium cation and the π-electrons of nanotubes [30], while MWCNTs are used to decrease the charge transfer resistance and improve the electrical conductivity at the interface between the GCE and the polycation-sensitive membrane [31]. Under zero current conditions, there is no potential response to protamine, due to the presence of the neutral lipophilic salt DNNS-TDDA in the polycation-sensitive membrane. When a constant cathodic current is applied, there is a net flux of polycation in the direction of the membrane phase, since protamine can electrostatically interact with DNNS in the membrane phase to form the cooperative ion pairs [32]. Additionally, in order to obtain the reversible potential response, protamine can be removed from the polycation-sensitive membrane by applying a controlled voltage at the open-circuit potential.

### 3.2. Optimization of Protamine Concentration

**Figure 3:**
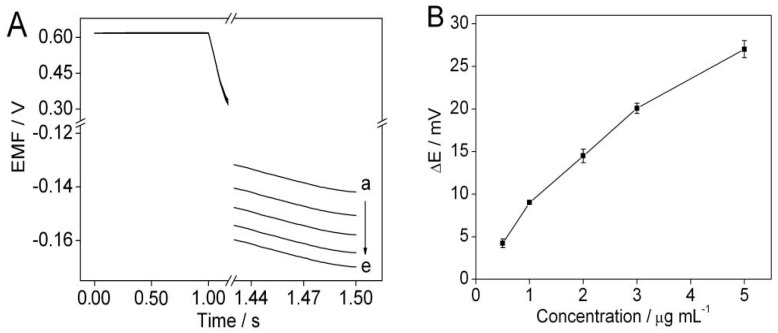
(**A**) Potentiometric responses of the solid-contact polycation-sensitive membrane electrode in 1.5 mL of 0.01 M sodium chloride solution with different protamine concentrations of (**a**–**e**) 5; 3; 2; 0.5; 0 μg·mL^−1^. (**B**) Plot shows potential changes over the protamine concentration range of 0–5 μg·mL^−1^. Error bars represent one standard deviation for three measurements.

Since protamine was used as an indicator to detect the change in the DNA concentration on the DNA nanostructure-modified magnetic beads induced by *V. alginolyticus*, the effect of protamine concentration was investigated. Figure 3A show s the potentiometric responses of the solid-contact polycation-sensitive membrane electrode under an external cathodic current of 5 μA with a duration of 1 s. It can be seen that the potential response of the polycation-sensitive membrane electrode in the presence of protamine is larger than that in the absence of protamine, which indicates that protamine is extracted into the polycation-sensitive membrane. Figure 3B shows the curve of the potential change of the membrane electrode vs. the concentration of protamine. When the concentration of protamine is 5 μg·mL^−1^, the potential change is up to 30 mV. Considering the subsequent electrostatic interaction between the DNA nanostructures and protamine and the sensitivity of the polycation-sensitive membrane electrode, 5 μg·mL^−1^ protamine was selected for further experiments.

### 3.3. Optimization of the Amount of the DNA Nanostructure-Modified Magnetic Beads

**Figure 4:**
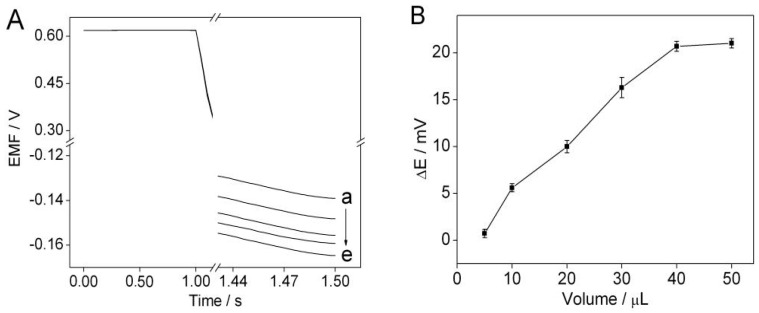
(**A**) Potentiometric responses of the solid-contact polycation-sensitive membrane electrode in 1.5 mL of 0.01 M sodium chloride solution with 5 μg·mL^−1^ protamine in the presence of DNA nanostructure-modified magnetic beads with different volumes of (**a**–**e**) 0; 5; 10; 30; 40 μL. (**B**) Plot shows the potential changes over the volume range of 0–50 μL for the DNA nanostructure-modified magnetic beads. Error bars represent one standard deviation for three measurements.

The influence of the amount of the DNA nanostructure-modified magnetic beads on the potential response to protamine of the solid-contact polycation-sensitive membrane electrode was investigated. As shown in Figure 4A, there is an obvious potential change in the presence of the DNA nanostructure-modified magnetic beads, which is due to the electrostatic interaction between the DNA nanostructures and protamine. Additionally, as shown in Figure 4B, the potential change increases as the amount of DNA nanostructure-modified magnetic beads increases up to 40 μL, and thereafter remains almost constant. Therefore, a volume of 40 μL was used as the amount of the DNA nanostructure-modified magnetic beads for further study.

### 3.4. The Detection of V. alginolyticus via Chronopotentiometry

**Figure 5:**
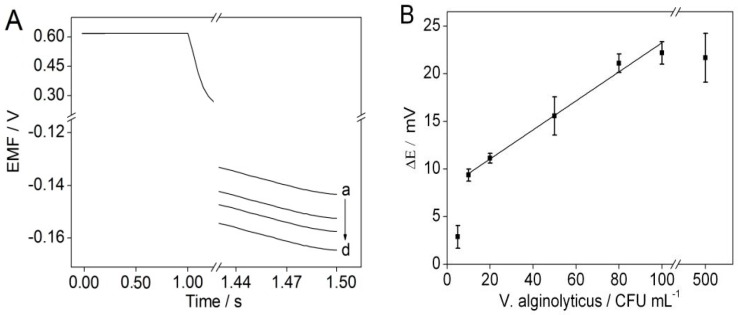
(**A**) Potentiometric responses of the solid-contact polycation-sensitive membrane electrode in 1.5 mL of 0.01 M sodium chloride solution with 5 μg·mL^−1^ protamine and 40 μL DNA nanostructure-modified magnetic beads in the presence of (**a**–**d**) 100; 50; 10; 0 CFU mL^−1^ *V. alginolyticus*. (**B**) Plot shows the potential changes over the *V. alginolyticus* concentration range of 5–500 CFU mL^−1^. Error bars represent one standard deviation for three measurements.

Under the optimal experimental conditions, the potentiometric responses to *V. alginolyticus* were tested by using the solid-contact polycation-sensitive membrane electrode via chronopotentiometry. As shown in Figure 5A, the potential response *V. alginolyticus* increases with increasing the concentration of *V. alginolyticus*, which indicates that the disassembly of the DNA nanostructures results in the decrease in the charge or DNA concentration on the surface of the modified magnetic beads. Figure 5B shows the calibration curve of the potentiometric aptasensing assay. It can be seen that the linear range for the proposed potentiometric aptasensing assay is 10–100 CFU mL^−1^, and the detection limit is 10 CFU mL^−1^.

### 3.5. Specificity Test

**Figure 6:**
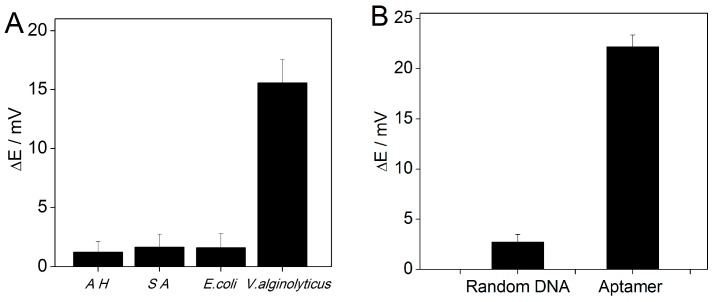
(**A**) Potential changes in the presence of 500 CFU mL^−1^ *Aeromonas hydrophila* (AH), *Staphylococcus aureus* (SA), *E. coli*, and 50 CFU mL^−1^ *V. alginolyticus*. (**B**) Potential responses to 100 CFU mL^−1^ *V. alginolyticus* using the aptamer and random DNA-based DNA nanostructures. Error bars represent one standard deviation for three measurements.

In order to test the specificity of the potentiometric aptasensing method for the detection of *V. alginolyticus*, four bacterial strains including *V. alginolyticus*, *E. coli*, *Staphylococcus aureus*, and *Aeromonas hydrophila* were tested. As shown in Figure 6A, no obvious potential changes are observed for these bacteria. This indicates that the proposed method for the detection of *V. alginolyticus* has a good specificity. Additionally, the influence of the random DNA on the potential response was also investigated (Figure 6B). The results indicate that the DNA nanostructures containing the random DNA would not induce any significant change in the potential response.

## 4. Conclusions

This work demonstrates a potentiometric aptasensing assay using signal amplification and magnetic separation strategies. The method is based on the DNA nanostructure-modified magnetic beads and the solid-contact polycation-sensitive membrane electrode for the reversible chronopotentiometric detection of *V. alginolyticus*. The proposed method shows a linear concentration range of 10–100 CFU mL^−1^ with a detection limit of 10 CFU mL^−1^, and a good specificity for the detection of *V. alginolyticus*. The proposed strategy can be used for the detection of other microorganisms by changing the aptamers in the DNA nanostructures.

## Figures and Tables

**Table 1:** Sequences of the oligonucleotides used in this work.

Oligonucleotide	Sequence
Aptamer DNA	5′-TCAGTCGCTTCGCCGTCTCCTTCAGCCGGGGTGGTCAGTAGGAGCAGCACAAGAGGGAGACCCCAGAGGG-3′ [3]
Capture DNA	5′-TTTTTCCCTCTGGGGTCTCCC-3′ (modified with biotin at 5′ terminal end)
H 1 DNA	5′-CGGCGAAGCGACTGACAAAGTCTAGTCGCT-3′
H 2 DNA	5′-TCAGTCGCTTCGCCGAGCGACTAGACTTTG-3′
Random DNA	5′-GAGTAGTTCGTG GCCTAG-3′
